# *Lactiplantibacillus plantarum* N-1 improves autism-like behavior and gut microbiota in mouse

**DOI:** 10.3389/fmicb.2023.1134517

**Published:** 2023-03-16

**Authors:** Zhongqing Qiu, Dongmei Luo, Heng Yin, Yajun Chen, Zhiwei Zhou, Jing Zhang, Linzhu Zhang, Jinrong Xia, Jiang Xie, Qun Sun, Wenming Xu

**Affiliations:** ^1^Chengdu University of Traditional Chinese Medicine, Chengdu, China; ^2^Chengdu Third People’s Hospital, Chengdu, China; ^3^Key Laboratory of Bio-resources and Eco-Environment, The Ministry of the Education, College of Life Sciences, Sichuan University, Chengdu, Sichuan, China; ^4^Key Laboratory of Birth Defects and Related Diseases of Women and Children, Ministry of Education, West China Second University Hospital, Sichuan University, Chengdu, China; ^5^Reproductive Endocrinology and Regulation Laboratory West China Second University Hospital, Sichuan University, Chengdu, China

**Keywords:** autism, maternal immune activation, *Lactiplantibacillus plantarum* N-1, gut microbiota, behaviors

## Abstract

**Introduction:**

The gut-brain axis has been widely recognized in autism spectrum disorder (ASD), and probiotics are considered to potentially benefit the rescuing of autism-like behaviors. As a probiotic strain, *Lactiplantibacillus plantarumN-1*(*LPN-1*) was utilized to investigate its effects on gut microbiota and autism-like behaviors in ASD mice constructed by maternal immune activation (MIA).

**Methods:**

Adult offspring of MIA mice were given *LPN-1* at the dosage of 2  ×  10^9^  CFU/g for 4  weeks before subject to the behavior and gut microbiota evaluation.

**Results:**

The behavioral tests showed that *LPN-1* intervention was able to rescue autism-like behaviors in mice, including anxiety and depression. In which the *LPN-1* treatment group increased the time spent interacting with strangers in the three-chamber test, their activity time and distance in the central area increased in the open field test, and their immobility time decreased when hanging their tails. Moreover, the supplementation of *LPN-1* reversed the intestinal flora structure of ASD mice by enhancing the relative abundance of the pivotal microorganisms of *Allobaculum* and *Oscillospira*, while reducing those harmful ones like *Sutterella* at the genus level.

**Discussion:**

These results suggested that *LPN-1* supplementation may improve autism-like behaviors, possibly *via* regulating the gut microbiota.

## 1. Introduction

Autism spectrum disorder (ASD) is a heterogeneous neurodevelopmental disorder consisting of three core symptoms: communication deficits, impaired sociability, and repetitive or restricted behavior (Lord et al., 2018). The incidence is higher in males than in females, with the ratio being closer to 3:1 (Loomes et al., 2017). ASD affects more than 1% of children in Western countries, while the prevalence in China is as high as 0.7% (Zhou et al., 2020), and the rate is on the rise due to the improvements in identification, screening, clinical assessment, and diagnostic testing (Genovese and Butler, 2020). However, effective treatments for ASD remain elusive, and the etiology is also unknown. The major contributing factors that have been studied include genetics, environmental factors, and health conditions (Lyall et al., 2017).

It is reported that ASD patients are often afflicted with gastrointestinal (GI) problems (Kohane et al., 2012; Vuong and Hsiao, 2017), including diarrhea/constipation, abdominal pain, and gastric reflux. The studies suggest that may be caused by the presence of different intestinal flora structures in people with ASD than in healthy ones (Williams et al., 2011; Vuong and Hsiao, 2017; Coretti et al., 2018; Wang et al., 2019). Recent studies have found that increased intestinal *Lactobacillus* and *Desulfovibrio* species in ASD patients are associated with the severity of ASD (Adams et al., 2011; Tomova et al., 2015). It has also been shown that *Bifidobacterium*, *Prevotella*, and butyric acid-producing bacteria are reduced and *Desulfovibrio*, *Clostridium*, and *Sutterella* are increased in ASD patients compared to healthy individuals (Zhang et al., 2018; Liu et al., 2019). Moreover, evidence from animal models indicates that specific gut microbial changes may result in clinical symptoms resembling ASD. Probiotics and prebiotics can alleviate behavioral deficits, inflammatory responses and intestinal flora dysbiosis in a prenatal valproic acid (VPA)-induced rodent model of autism (Adıgüzel et al., 2022). What’s more. The ecological dysbiosis of the intestinal microbiota in ASD mice was found to be driven mainly by alterations in specific operational taxonomic units (OTUs) of the bacterial classes *Clostridium* and *Bacteroides fragilis*, and treatment with *B. fragilis* was found to improve autism-related symptoms by improving intestinal flora and intestinal barrier function (Hsiao et al., 2013). These suggest that gut microbiota regulates normal host physiology, metabolism, nutrition, and brain function. Increasing research reveals the ability of the gut microbiota to signal across the so-called microbiota-gut-brain axis. A recent study shows that oral probiotics prevent maternal immune activation (MIA)-induced increases in IL-6 and IL-17A levels in both maternal serum and fetal brains, parvalbumin-positive (PV+) neuron loss, and the decrease in γ-aminobutyric acid levels in the prefrontal cortex of adult offspring (Wang et al., 2019). Clinical studies have also demonstrated that the use of probiotics and fructo-oligosaccharides can ameliorate ASD symptoms, including hyperserotonergic states and dopamine metabolism abnormalities, by altering the gut microbiota and increasing the amount of short-chain fatty acids (SCFAs) and serotonin (Wang et al., 2020). Two other studies showed that probiotics could improve social and self-grooming behaviors as well as intestinal permeability in the BTBR T^+^ Itpr3^tf^/J (BTBR) Mouse Model of ASD (Nettleton et al., 2021; Pochakom et al., 2022).

Although previous studies showed that probiotics had the potential to reduce GI distress in individuals with ASD (Sanctuary et al., 2019), little was known about their effects on ASD behavior directly. The strain of *Lactiplantibacillus plantarum* N-1 (*LPN-1*; CGMCC NO. 15463), isolated from traditional cheese in Daocheng County, Sichuan Province by our laboratory before, is a probiotic strain. *In vivo* and *in vitro* experiments have shown that *LPN1* has multiple probiotic functions, including acid- and bile salt-tolerant biology, the ability to modulate intestinal flora structure by producing multiple SCFAs, especially butyric acid, enhance intestinal barrier function, and reduce inflammation levels (Liu et al., 2017, 2021; Wei et al., 2021; Tian et al., 2022, 2023). Therefore, we hypothesized that *LPN-1*, with broad-spectrum intestinal flora improvement effects, may improve anxiety-like behavior in ASD mice by improving their gut microbiota. Therefore, in this study *LPN-1* intake was examined for its improvement of autistic-like behavior and its effect on the gut microbiota in the ASD mice model.

## 2. Materials and methods

### 2.1. Maternal immune activation rodent care and intervention

**Figure 1:**
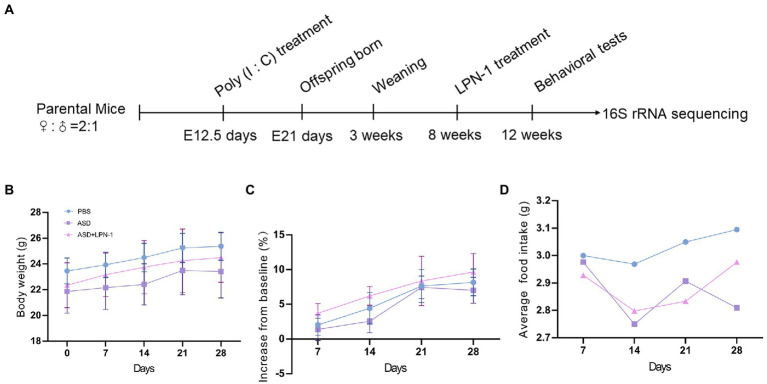
Schematic of experimental design **(A)**. Body weight over time **(B)**. Body weight gain (% increase from baseline) **(C)**. Average daily food intake **(D)**.

This study was approved by the Animal Ethics Committee of West China Second University Hospital, Sichuan University (2020–035). There is a link between viral infection during pregnancy and an increased incidence of ASD in the child (Choi et al., 2016). Therefore, MIA is widely used in ASD research (Naviaux et al., 2013, 2014; Vuillermot et al., 2017; Minakova et al., 2019; Fujita et al., 2020; Xu et al., 2021; Tartaglione et al., 2022). We used a mouse model subjected to MIA, which was constructed by injecting pregnant mothers with poly (I:C; 20 mg/kg) on embryonic day 12.5, while the control group was injected with phosphate-buffered saline (PBS). Adult male offspring of MIA mice were randomized to (1) PBS; (2) ASD; (3) ASD + LPN-1 (2 × 10^9^ CFU/g Also called *LPN-1* group) administered through food for 4 weeks as shown in Figure 1A. Body weight and food intake were measured weekly. The animals [four mice/cage, and no single cage rearing for animal welfare (National Research Council Committee, 2011)] were housed in the Medical Laboratory Animal Center of West China Second University Hospital, Sichuan University, under SPF conditions, with a relative humidity of about 50%, temperature control of 22–25°C, adequate food and water, and 12/12 h fixed light cycle.

### 2.2. Behavior tests

The test mice were placed in the behavioral room 5 days in advance to acclimate to the environment. During the behavioral period, the testers tried to keep the color of their clothing the same. At about 14:00–18:00 every day, the mice were stroked on the experimenter’s hand at a fixed time, 5 min each time for each mouse, to reduce nervousness and familiarize them with the experimenter. Mice were rested for 3–5 days before the next behavioral test.

#### 2.2.1. Three-chamber test

A three-chamber device was used to test the social communication abilities of different groups of mice. The apparatus consisted of three Plexiglas chambers (60 × 40 × 22 cm), with the side chambers each connected to the middle chamber by a corridor (10 × 5 cm). The sociability of ASD mice was tested using a three-chambered device for three consecutive 10 min phases. During the first phase, mice were habituated to the three chambers for 10 min. In the second phase, two wire cages were introduced to the side chambers: one wire cage was empty, while the other was set up with unfamiliar mouse of the same sex and age which had no previous contact (stranger 1). The testing mouse was placed in the middle chamber, and the amount of time spent around each cage (stranger 1 or empty) was measured. Finally, an unfamiliar mouse (stranger 2) was placed in one of the side chambers, and a familiar mouse (stranger 1) was placed in the other side chamber. The testing mouse was free to explore the mouse from the previous sociability test (stranger 1), and the novel mouse (stranger 2). The time spent in each chamber was recorded. Social behaviors were analyzed using a social behavioral analysis system (BW-Social LAB, Shanghai Biowill Co., Ltd.). The Plexiglas chamber was sterilized with 75% ethanol and wiped dry using paper towels between animal tests.

#### 2.2.2. Open-field test

An open-field experiment device (40 × 40 × 40 cm) was used to detect the mice’s anxious behavior. The test was performed using a method similar to a previous report (Katano et al., 2018). Before the test, the mice were placed in the device for 5 min, and then their behavior was recorded for 10 min. During the experiment, a curtain was used to completely isolate the experimental device from the external environment to avoid noise affecting the behavior of mice. Anxious behaviors were analyzed using a social behavioral analysis system (BW-Social LAB, Shanghai Biowill Co., Ltd.). The Plexiglas chamber was sterilized with 75% ethanol and wiped dry using paper towels between animal tests.

#### 2.2.3. Novel object recognition test

The test was performed in an open field arena (40 × 40 × 40 cm). The novel object recognition test consisted of two stages. During a 10 min acquisition phase, the animals were placed at the center of the arena in the presence of two identical objects (6 × 6 × 6 cm). After 2 h, a 5 min retrieval phase was conducted, and one of the two familiar objects was replaced by a novel object (5 × 5 × 5 cm). The time spent exploring familiar and novel objects was recorded and analyzed. Exploration time is defined as the action of pointing the nose toward an object, at a maximum distance of 2 cm or touching it (Ennaceur and Delacour, 1988).The Plexiglas chamber was sterilized with 75% ethanol and wiped dry using a paper towel between animal tests. The “discrimination index” was calculated as follows: [(novel object time)/(novel object time + familiar object time)].

#### 2.2.4. Tail suspension test

The tail suspension test is a behavioral test commonly used to detect depression in mice. We used specially manufactured tail suspension boxes made of plastic with the dimensions 55 cm height × 15 cm width × 11.5 cm depth. The mouse was suspended in the middle of this compartment, and the width and depth were sufficiently large so that the mouse could not make contact with the walls. The approximate distance between the mouse’s nose and the apparatus floor was 20–25 cm. The resultant behavior was recorded by a video camera for 6 min. The behavior was later analyzed to determine the total duration of immobility; the total amount of time during which each mouse remained immobile was recorded in seconds. The Plexiglas chamber was sterilized with 75% ethanol and wiped dry using paper towels between animal tests.

### 2.3. Histopathological examinations

At the end of all behavioral experiments, the liver, kidney, and colon tissue were carefully removed and followed by phosphate-buffered saline wash. Then they were fixed in 10% phosphate-buffered formalin for 24 h. After dehydration, they were embedded in paraffin, the paraffin blocks were cut at 5 μm using a microtome, and the deparaffinized tissue slices were subjected to Masson and hematoxylin eosin (H&E) for histological examination.

### 2.4. The 16S rRNA gene sequencing

Fresh fecal samples were collected from the rectum at the end of the experiment and stored at −80°C. DNA was extracted and quantified by Nanodrop and the quality of DNA extraction was detected by 1.2% agarose gel electrophoresis (Nazhad and Solouki, 2008). The V3-V4 region of the bacterial 16S rRNA genes was amplified by polymerase chain reaction with primers 338F 5′-ACTCCTACGGGAGGCAGCA-3′ and 806R 5′-CGGACTACHVGGGTWTCTAAT-3′ (Wei et al., 2021). The PCR-amplified product was purified, quantified, and sequencing libraries were prepared using Illumina’s TruSeq Nano DNA LT Library Prep Kit. The original sequences that passed the initial quality screening were subjected to the library and sample partitionin. Sequence denoising was performed according to the QIIME2 dada2 analysis process to obtain amplicon sequence variants (ASV). α-diversity and β-diversity were finally analyzed. And raw sequences have been uploaded to the NCBI database, No. PRJNA916455.

### 2.5. Statistical analysis

All data were expressed as mean ± SEM. Statistical analyses were performed using GraphPad Prism (version 8.0.2). The results were performed using two-way analysis of variance (ANOVA) or one-way ANOVA. *p*  <  0.05 was considered statistically significant.

## 3. Results

### 3.1. *LPN-1* improves social tests, reduce anxious and depression behavior in ASD mice

**Figure 2:**
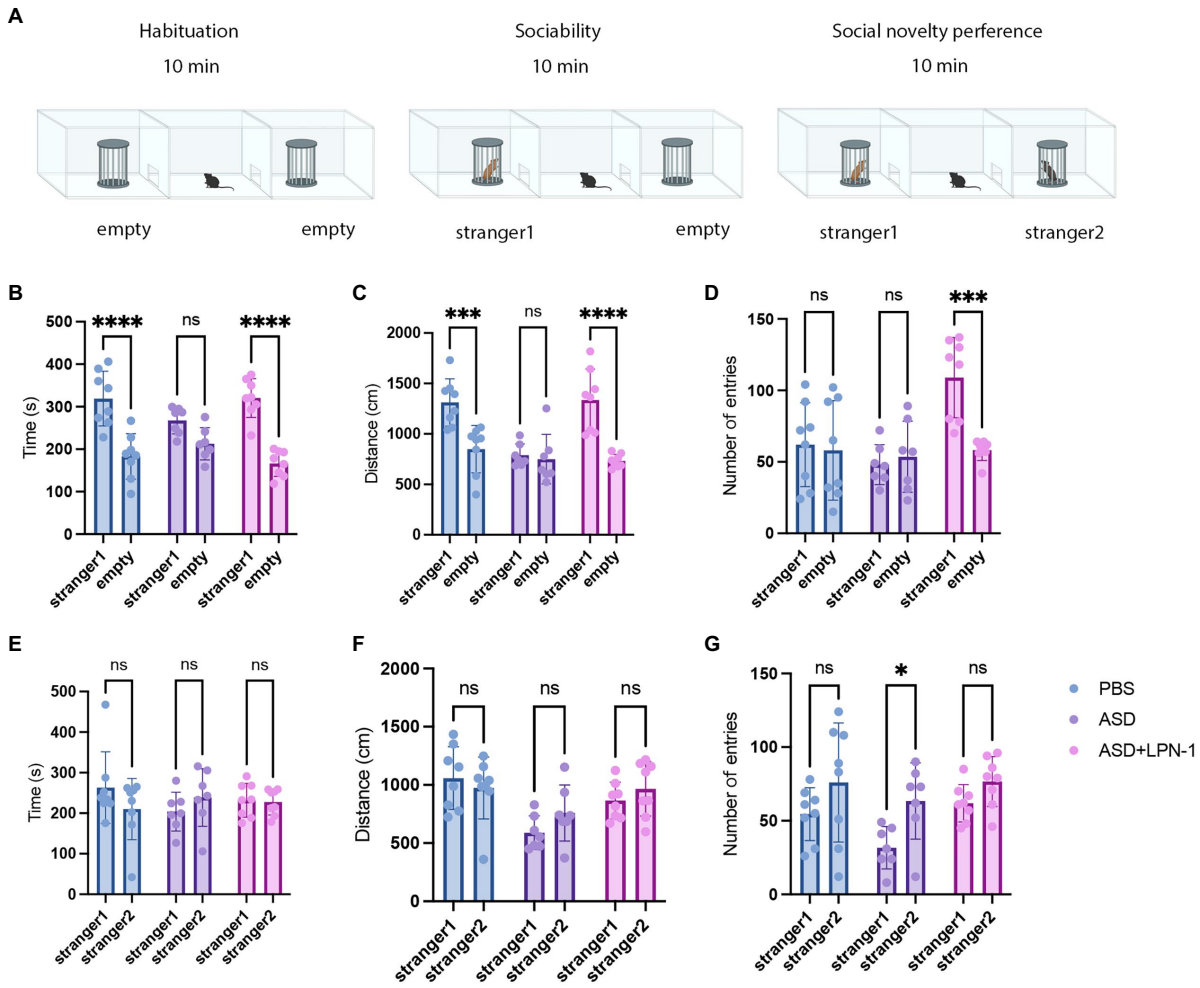
Sociability and social novelty in mice, phosphate-buffered saline (PBS) (*n* = 8), ASD (*n* = 7), ASD + LPN-1 (*n* = 8). **(A)** Schematic representation of the three-chamber social interaction test. Sociability test **(B–D)**: Time spent near the wire cage with stranger 1 **(B)**, distance walked around stranger 1 **(C)**, and number of entries **(D)**. Social novelty preference test **(E–G)**: Time spent near the wire cage with stranger 2 **(E)**, distance walked around stranger 2 **(F)**, and number of entries **(G)**. ^*^p < 0.05, ^**^p < 0.01, ^***^p < 0.001, ^****^p < 0.0001.

We used the three-chamber social test to determine the social–behavior abnormality (Figure 2A). We compared the time and distance spent in the chamber containing stranger 1 and the empty chambers. Mice in the PBS group (*n* = 8) spent more time and traveled a greater distance with stranger 1, whereas mice in the ASD group (*n* = 7) spent less time (Figure 2B) and traveled a shorter distance (Figure 2C), indicating social interaction deficits. In contrast, mice treated with *LPN-1* spent more time with stranger 1 (*p* < 0.0001; Figure 2B) while spending significantly less time in empty chambers, indicating that the social preference index was significantly altered. We also found a significant increase in the number of entries in the ASD + LPN-1group (*n* = 8; *p* < 0.001; Figure 2D). There was no significant difference in the social time (Figure 2E) among the groups or in the social distance (Figure 2F), but the number of entries to stranger 2 significantly increased in the ASD group (*p* < 0.05; Figure 2G). The results showed that *LPN-1* could effectively rescue part social deficiency caused by poly (I:C) treatment during pregnancy.

**Figure 3:**
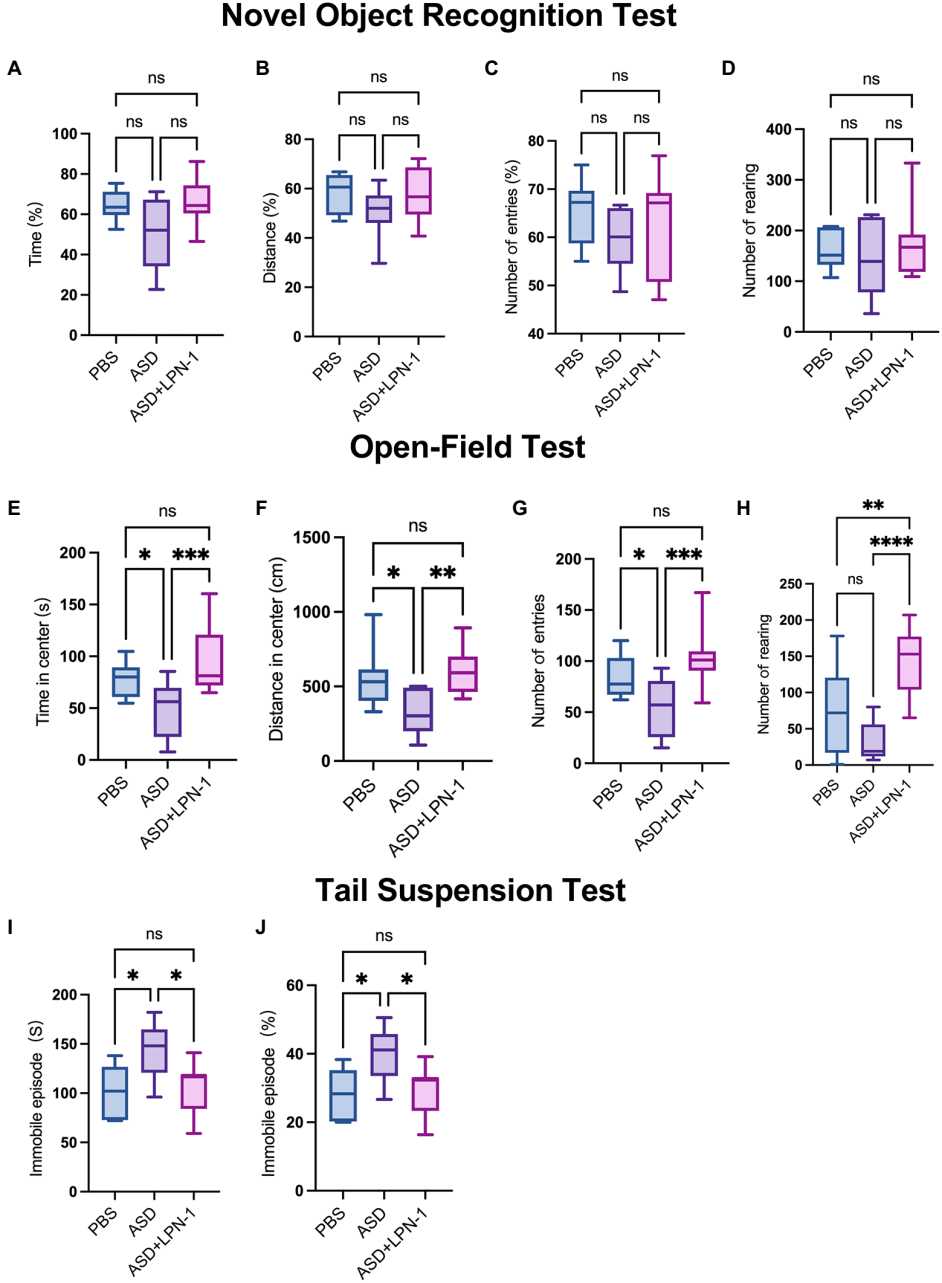
(1) Novel object recognition test, phosphate-buffered saline (PBS) (*n* = 6), autism spectrum disorder (ASD) (*n* = 6), ASD + LPN-1 (*n* = 8). Experimental schedule showing the different phases of the NOR familiarization (10 min), and test (5 min). Discrimination index of time spent around the novel object **(A)** and the distance **(B)** or the number of entries **(C)**. Total number of rearing **(D)**. (2) Open-field test, PBS (*n* = 12), ASD (*n* = 9), ASD + LPN-1 (*n* = 12). Time spent in center **(E)**. Distance traveled in the center **(F)**. Number of entries **(G)**. Number of rearing **(H)**. (3) Tail suspension test, PBS (*n* = 6), ASD (*n* = 6), ASD + LPN-1 (*n* = 11). Graphs show the total time spent immobile **(I)** and the proportion of immobility time **(J)**. ^*^p < 0.05, ^**^p < 0.01, ^***^p < 0.001, ^****^p < 0.0001.

The novel object recognition (NOR) test is a relatively fast and efficient means of testing different phases of learning and memory in mice. In the NOR paradigm, we found a slight decrease in the time, distance, and the number of entries to novel objects explored by ASD mice. However, there were no significant differences among the three groups in the time spent around the novel object (Figure 3A), the distance (Figure 3B), or the number of entries (Figure 3C) and rearing (Figure 3D). Thus, in terms of cognitive performance, mice in the PBS (*n* = 6), ASD (*n* = 6), and ASD + LPN-1 (*n* = 8) groups did not show significant differences. However, *LPN-1* intervention tended to increase the ability of ASD mice to explore new things, and may reach significant levels if the duration of *LPN-1* intervention increases.

We then performed the open-field test for a total duration of 10 min to detect anxious behavior in mice. In this test, the open-field trials present a conflict between the innate drive to explore a new environment and personal safety (Crawley, 2008). The longer time spent in the central area of the open field, the more distance traveled in the central area, and the more entries to the center indicate less anxious behavior in the mice (Crawley et al., 1997). As shown in Figures 3E,F, the PBS group (*n* = 12) spent more time and traveled longer distances in the center compared to the ASD group (*n* = 9). The results showed that the ASD group spent less time in the center, walked shorter distances, and entered the central area fewer times (Figure 3G), suggesting that the ASD group had obvious anxiety behavior. However, after supplementation with *LPN-1*, there was no significant difference between the PBS group and the ASD + LPN-1 group (*n* = 12), indicating that the anxiety behavior of the mice was reduced. However, the increased rearing in the central area of the ASD + LPN-1 group indicated an increase in repetitive behavior (Figure 3H). We used the tail suspension test to analyze depression-like behavior, as previously described (Umemura et al., 2017; Ueno et al., 2019). In the tail suspension test, the ASD group (*n* = 6) showed significantly increased immobility (*p* < 0.05; Figures 3I,J), indicating enhanced depressive-like behavior. Immobility time decreased after *LPN-1* supplementation, and there was no significant difference in immobility time between the PBS group (*n* = 6) and the ASD + LPN-1 group (*n* = 11), indicating that *LPN-1* may reduce the depressive behavior of mice. Together, all the battery of behavior tests indicate that *LPN-1* may improves social tests, reduce anxious and depression behavior in ASD mice model.

### 3.2. *LPN-1* conduce no harm to the organ tissues of ASD mice in this study

**Figure 4:**
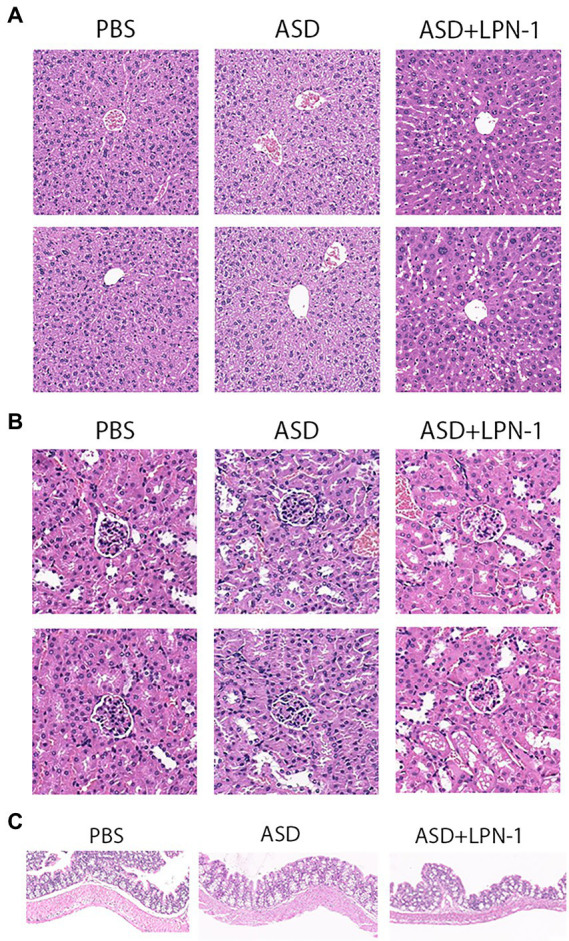
H&E staining showing histological cell morphology and inflammatory changes. H&E used to observe liver tissue **(A)**, the cortex, including glomeruli, and renal interstitium are shown **(B)**, and sections stained with H&E to assess the mucous membrane appearance of the colon **(C)**.

We recorded the body weight and food intake of the animals on a weekly basis during the experiment (Figures 1B–D). All the mice were sacrificed at the end of the behavioral test, and their liver, kidney, and colon tissues were excised to assess the safety of *LPN-1*. H&E staining revealed that regular hepatic sinusoidal structure and clear hepatic lobules were observed in liver tissues, and cell edema, inflammatory cell infiltration, and severe intrahepatic hemorrhage were not observed in the three groups of mice (Figure 4A). The morphology and organization of renal tissues in the sham group were normal; vacuolar degeneration in renal tubular epithelial cells, detachment of renal tubular epithelial cells, and infiltration of inflammatory cells were not observed (Figure 4B). As shown in Figure 4C, the colonic structure of the three groups of mice was intact, and the intestinal glands were well arranged. Moreover, infiltration of inflammatory cells was not observed in the lamina propria mucosa and muscular layer.

### 3.3. *LPN-1* modulates the gut microbiota of ASD mice

**Figure 5:**
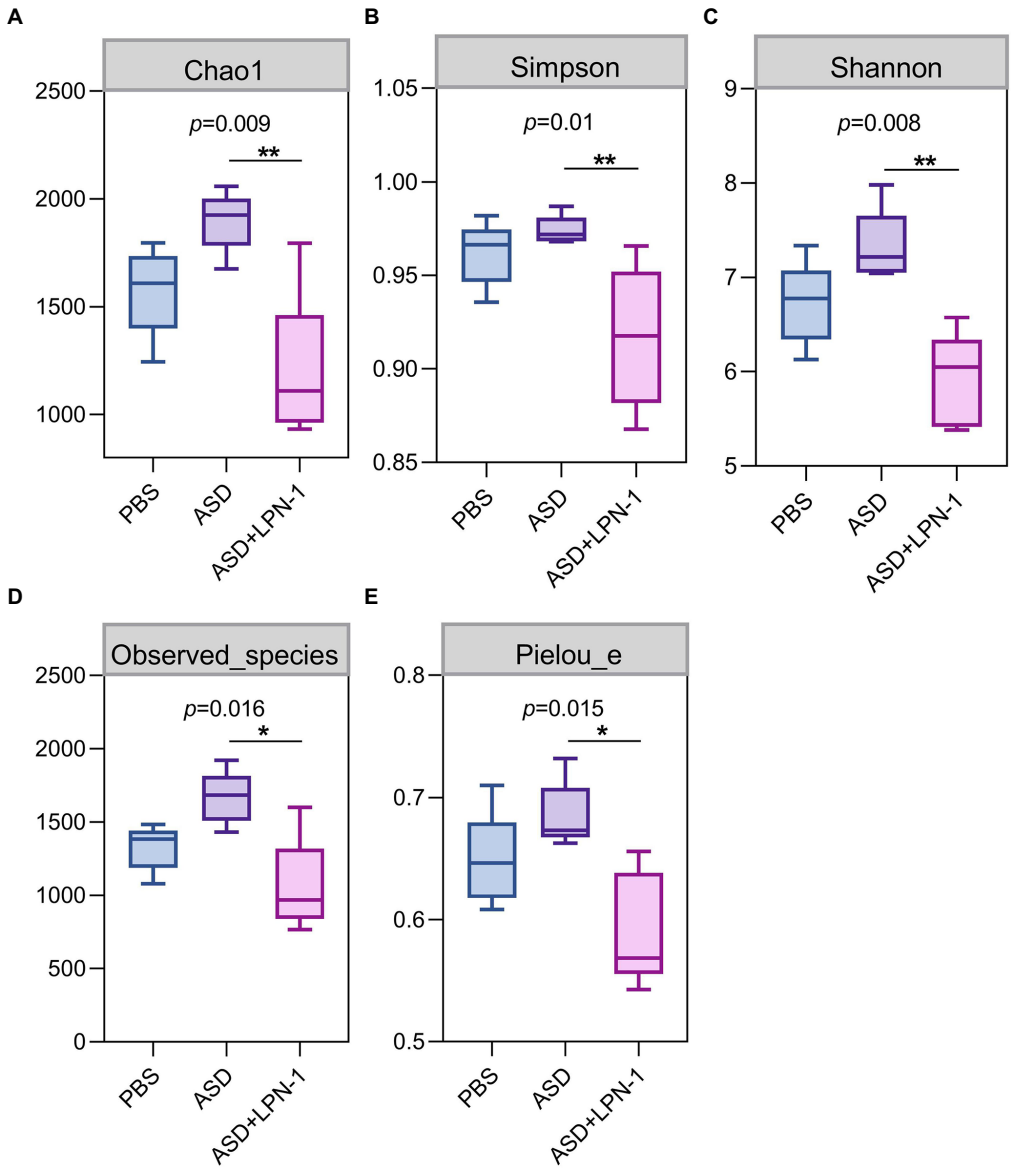
The alpha diversity assessed by Chao1 **(A)** Simpson **(B)** Shannon **(C)** Observed_species **(D)** Pielou_e index **(E)**. Statistically significant differences among groups were determined per the Kruskal-Wallis test. *n*=5 per group. *p < 0.05. **p < 0.01.

**Figure 6:**
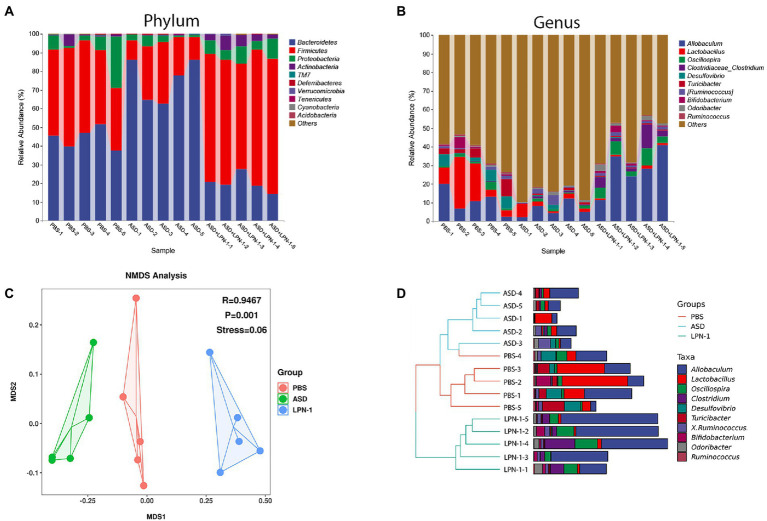
Histogram of species distribution at the phylum **(A)** and genus **(B)** levels revealed by 16S rRNA sequencing. NMDS analysis based on weighted_unifrac_distance among phosphate-buffered saline (PBS), autism spectrum disorder (ASD), and ASD + LPN-1 groups **(C)**. Hierarchical clustering analysis **(D)**.

**Figure 7:**
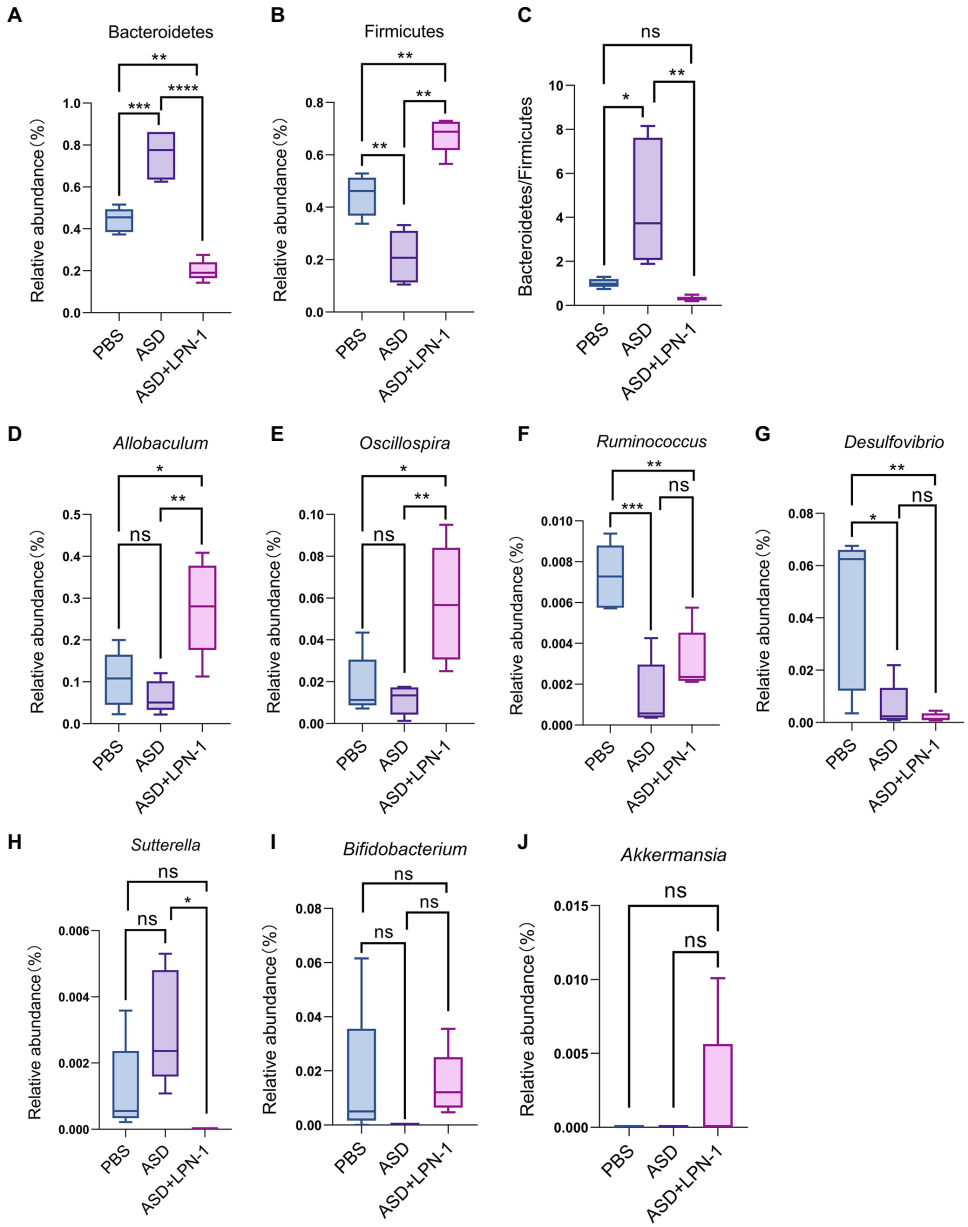
The relative abundance of gut microbiota at the phylum **(A–C)** and genus **(D-J)** level. ^*^p < 0.05, ^**^p < 0.01, ^***^p < 0.001, and ^****^p < 0.0001.

Figures 5–7 showed the results of species annotation analysis at the phylum and genus levels in three groups revealed by 16S rRNA sequencing. The alpha diversity indexes of Chao1, Pielou_e, and Shannon characterized significant differences in microbial populations among the PBS (*n* = 5), ASD (*n* = 5), and *LPN-1* (*n* = 5) groups (*p* < 0.05). Multiple alpha diversity metrics of evenness, diversity and richness in ASD mice were higher than in PBS mice, but *LPN-1* supplementation decreased those alpha diversity indexes (Figure 5). According to the Non-metric Multidimensional scaling (NMDS), the apparent separation of microbial population structures between the ASD group and *LPN-1* group was illustrated (Figure 6C). Furthermore, hierarchical clustering analysis of the unweighted pair-group method with the arithmetic mean (UPGMA) showed that *LPN-1* group clustered differently between ASD and PBS groups (Figure 6D). This indicated that the three groups have different gut microbial compositions. In addition, the reduced alpha diversity of the *LPN-1* group suggests that a dominant genus may have emerged and occupied the ecological niche of gut microflora. Therefore, the phylum and genus levels of gut microbiota in each group were further analyzed.

Firmicutes and Bacteroidetes were the most predominant phyla in the gut bacteria of mice and abundant in all samples accounting for almost 90% (Figure 6A). Compared to the PBS group, Bacteroidetes increased and Firmicutes decreased in the ASD group. However, the *LPN-1* supplementation reversed this appearance (Figures 7A,B). And a significantly lower Bacteroidetes/Firmicutes ratio was shown in the *LPN-1* group compared to the ASD group (*p* < 0.01; Figure 7C). In addition, the results at the genus level showed that the relative abundance of *Allobaculum* was found to be more than 3-fold elevated after *LPN-1* intervention, becoming the absolute dominant group of intestinal microorganisms in the treated group of mice (Figure 6B). To further illustrate the significance of the differences, a one-way ANOVA analysis was performed on partial genera. The results showed that the intervention of *LPN-1* significantly elevated the abundance of beneficial bacteria including *Allobaculum* and *Oscillospira* (*p* < 0.01; Figures 7D,E) in the intestinal flora of ASD mice, as well as *Ruminococcus* (Figure 7F), *Bifidobacterium* (Figure 7I) and *Akkermansia* (Figure 7J) in spite of no significance yet. Further calculations showed that *Allobaculum* was elevated from 14.33 to 62.04% after *LPN-1* intervention compared with the model group. In addition to increasing the variety of probiotic bacteria, we observed that *LPN-1* treatment also significantly suppressed the abundance of the harmful bacterium *Sutterella* (*p* < 0.05; Figure 7H), and *Desulfovibrio* (Figure 7G) showed a decreasing trend in the *LPN-1* group.

## 4. Discussion

Neurodevelopmental diseases represented by ASD cause a huge medical burden to patients’ families and the whole society. Although the etiology is still unclear, infection and inflammation during pregnancy are considered to be key causes of ASD (Modabbernia et al., 2017). In animal models, poly (I:C) injection during pregnancy results in increased release of local cytokines, including IL-17a (Choi et al., 2016), which can recapitulate the key symptoms of ASD and be used to examine the efficacy of the candidate remedies, especially in MIA-associated ASD. Previous studies have found that probiotic supplementation in animal models can improve social deficits in mice with ASD (Sgritta et al., 2019) and improve anxiety-like behavior and elevate hippocampal BDNF levels in mice with low-grade intestinal inflammation (Bercik et al., 2010, 2011). Meanwhile, clinical studies have found that probiotic supplementation can reduce anxiety and depression behaviors and ameliorated the opposition and defiance behaviors of children with ASD (Liu et al., 2019; Kong et al., 2021). These studies only found the effects of probiotics on improving social interaction and alleviating anxiety, and did not find any negative effects, nor did they perform 16 s gene sequencing. In the present study, we found that *LPN-1* supplementation improved social and anxiety-like behaviors as well as depressive behavior, and that *LPN-1* intervention tended to increase the ability of ASD mice to explore new things. In contrast, repetitive behaviors have increased after *LPN-1* intervention.

The present results showed that *LPN-1* intervention significantly altered the intestinal flora structure of the ASD mice. The alpha diversity analysis revealed a significant decrease (*p* < 0.05) in the abundance, diversity, and homogeneity of the gut microbiome composition in all three groups, which may be due to the process of constructing the autism model led to an increase in the species and abundance of conditionally pathogenic bacteria in the intestine of the mice, and the *LPN-1* intervention resulted in antagonism between microorganisms reduced the species and abundance of conditionally pathogenic bacteria, leading to an overall decrease. Similar results were seen in a study related to autism (Wan et al., 2021), which measured gut microbes in children with autism and showed that gut microbial abundance was significantly higher in children with autism than in age-matched normal children. Treatment with *LPN-1* helped to restore the gut microbes of autistic mice to a similar structure to those of normal mice at the phylum level, including elevating the abundance of Bacteroidetes and reducing the abundance of Firmicutes. In addition, the ratio of gut microbial Bacteroidetes/Firmicutes in autistic mice was significantly different from that of normal individuals. Several publications have demonstrated that the ratio of Bacteroidetes/Firmicutes in the gut bacteria of children with ASD was significantly increased compared to normal subjects (Kang et al., 2017; Coretti et al., 2018; Zhang et al., 2018). The results of the present study are consistent with previous reports, and the ratio of Bacteroidetes/Firmicutes was significantly reduced compared to the model group by *LPN-1* treatment (*p* < 0.01).

In addition, analysis of gut microbial 16 s sequencing results revealed that *LPN-1* significantly increased the abundance of the probiotics *Allobaculum* and *Oscillospira* (*p* < 0.01) and decreased *Sutterella* (*p* < 0.05) at the genus level. Previous studies have shown that in ASD mice, *Allobaculum* abundance was significantly decreased and that GW4064 (a farnesoid X receptor agonist) restored the abundance of *Allobaculum* and improved autism (Liu et al., 2022). Moreover, it has been shown that *Allobaculum* is highly correlated with depression in mice, and this study showed a positive association between *Allobaculum* and neurotransmitter norepinephrine secretion in mice by correlation analysis (Wu et al., 2020; Xia et al., 2021). In conclusion, *Allobaculum* may be positively correlated with the treatment of various neurological diseases and showed a correlation with neurotransmitter secretion and neuronal development. Therefore, We supposed *LPN-1* may affect the neurodevelopment of the organism by increasing the abundance and metabolism of the *Allobaculum* in the intestine to improve autism-related symptoms. The correlation between intestinal flora and clinical characteristics of children with ASD revealed that *Oscillospira* was negatively correlated with the Total Childhood Autism Rating Scale score and *Oscillospira* was significantly increased after *LPN-1* intervention in our study (*p* < 0.01) (Chen et al., 2021). More surprisingly, the probiotics *Bifidobacterium* and *Akkermansia* occurred from absent to present in the intestine of ASD mice after *LPN-1* intervention. As far as why it did not reach a significant increase, we speculate the time of one-month intervention is a bit short and the intestinal flora structure has not yet been achieved much well. Therefore, our subsequent animal experiments as well as clinical experiments will increase the intervention time of *LPN-1* to make it reach the best condition.

In contrast, there was no *Sutterella* in the *LPN-1* group. *Sutterella* was one of the most important sources of lipopolysaccharide LPS, which could affect intestinal permeability and lead to an increase in plasma LPS concentration, triggering chronic low-grade inflammation in the organism. The relative abundance of *Sutterella* was higher in the intestine of children with ASD compared to normal children (Kang et al., 2017). A study shows that *Sutterella* was the predominant flora in ileal and cecum biopsies of children with autistic children with gastrointestinal dysfunction (AUT-GI) (Williams et al., 2012). In animal experiments, again with results similar to human studies, it was shown that the abundance of *Sutterella* in the colon of the offspring of autistic mice was significantly higher than that of normal mice (Sharon et al., 2019). Therefore, our findings suggested that the intake of probiotic *LPN-1* not only increased the abundance of probiotics, but also reduced harmful bacteria, improved the structure of intestinal flora, and facilitates its healthy development.

Our results suggested that probiotics may improve ASD by affecting gut flora, however it was inconsistent with the results of another study which, after correlating fecal macrogenomic and phenotypic data from children with ASD at a mean age of 8.7 years, concluded that it was not differences in gut flora that caused ASD, but the dietary preferences of children with ASD that caused the differences in gut flora (Yap et al., 2021). This discrepancy between our study and the results of that study, may be due to the fact that the data collection time of that study mostly spanned a critical period of neurodevelopment [before the age of three is an essential stage of human brain development (Cody et al., 2017)], and that the symptoms of these children were generally mild and perhaps not representative of the typical autistic population, not to mention denying the driving role of the flora. Of course, our ongoing experiments are proposed to elucidate how the probiotic *LPN-1* improves autistic symptoms through the gut-brain axis (e.g., enterobacterial metabolites, intestinal permeability, blood–brain barrier, etc.), and we hope that our research can scientifically and objectively guide the public’s perception of the relationship between autism and intestinal flora. However, there are some limitations in this study as well. We examined the effect of *LPN-1* in ASD mice, but not in normal mice. The combination of *LPN-1* with other probiotics or therapeutic drugs and the duration of effective treatment deserved further study. Therefore, much studies in the prevention of neurological diseases like ASD by combining probiotics with other drugs are needed. In addition, our study was conducted only in adult c57BL/6 male ASD mice, and female ASD mice were not included. Results may also be different in mice from other disease backgrounds, other age groups and other strains, like juvenile mice with unstable and immature microbiome structures. Probiotics act slowly and require a long-term continuous intervention to achieve a stable intervention, whereas in our study we only intervened for 4 weeks after the weaning period. For the sake of animal welfare, the mice in our experiments were not housed singly in a single cage and the final conclusions may need to be treated with caution.

## 5. Conclusion

We demonstrated that *LPN-1* improved autism-like social phobic and depressive behavior in mice from a poly (I: C)-induced maternal immune activation model. The vital role of *LPN-1* in increasing probiotic bacteria, including *Allobaculum* and *Oscillospira*, and decreasing the harmful ones of *Sutterella* in the gut microbiota was also highlighted, indicating the efficacy of *LPN-1* intervention in the animal model. Further research on how *LPN-1* affects neurologically related autism-like behavior *via* the gut-brain axis is under process. This study may provide new insight into the development of psychobiotics to ameliorate the autism-associated neurological disorders.

## Data availability statement

The data presented in the study are deposited in the NCBI repository, accession number PRJNA916455.

## Author contributions

ZQ conceived and designed the work that led to the submission. DL joined the microbial experiments, data analysis and writing. HY, YC, JZ, LZ, and JinX conducted the lab work. ZZ joined the data analysis. JiaX, WX, and QS managed project design and process. All authors contributed to the article and approved the submitted version.

## Funding

The authors gratefully acknowledge the financial supports by the Science and Technology Plan Project of Sichuan Province (2021YJ0170); Regional Cooperation Project (Sichuan-Guangdong) of Sichuan Science and Technology Programs (2022YFQ0100); and the Chengdu Science and Technology Project (2019-YF05-00498-SN).

## Conflict of interest

The authors declare that the research was conducted in the absence of any commercial or financial relationships that could be construed as a potential conflict of interest.

## Publisher’s note

All claims expressed in this article are solely those of the authors and do not necessarily represent those of their affiliated organizations, or those of the publisher, the editors and the reviewers. Any product that may be evaluated in this article, or claim that may be made by its manufacturer, is not guaranteed or endorsed by the publisher.
